# Ethyl 4-({1-[2-(4-bromo­phen­yl)-2-oxo­eth­yl]-1*H*-1,2,3-triazol-4-yl}meth­oxy)-8-(trifluoro­meth­yl)quinoline-3-carboxyl­ate

**DOI:** 10.1107/S1600536812046417

**Published:** 2012-11-24

**Authors:** Arun M. Islor, B. Garudachari, K. N. Shivananda, Thomas Gerber, Eric Hosten, Richard Betz

**Affiliations:** a National Institute of Technology-Karnataka, Department of Chemistry, Medicinal Chemistry Laboratory, Surathkal, Mangalore 575 025, India; b Technion Israel Institute of Technology, Schulich Faculty of Chemistry, Haifa, 32000, Israel; c Nelson Mandela Metropolitan University, Summerstrand Campus, Department of Chemistry, University Way, Summerstrand, PO Box 77000, Port Elizabeth, 6031, South Africa

## Abstract

The title compound, C_24_H_18_BrF_3_N_4_O_4_, is a 1,2,3-triazole derivative featuring, among others, a quinoline-derived substituent. In the crystal, C—H⋯O, C—H⋯N and C—H⋯F contacts connect the mol­ecules into a three-dimensional network. The shortest centroid–centroid distance between two aromatic systems is 3.896 (2) Å and is found between the two different six-membered rings of the quinoline scaffold in neighbouring mol­ecules.

## Related literature

For background to the industrial importance of heterocyclic compounds, see: Isloor *et al.* (2009 ▶); Vijesh *et al.* (2011 ▶); Ruanwasa *et al.* (2010 ▶). For pharmacological properties of quinoline-derived compounds, see: Chen *et al.* (2004 ▶); Kaur *et al.* (2010 ▶); Bekhit *et al.* (2004 ▶). For graph-set analysis of hydrogen bonds, see: Etter *et al.* (1990 ▶); Bernstein *et al.* (1995 ▶).
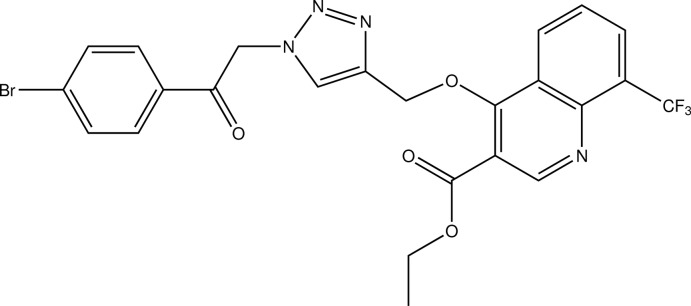


## Experimental

### 

#### Crystal data

C_24_H_18_BrF_3_N_4_O_4_
*M* *_r_* = 563.33
Monoclinic, 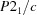

*a* = 5.2809 (2) Å
*b* = 24.5131 (10) Å
*c* = 18.3517 (7) Å
β = 99.643 (1)°
*V* = 2342.08 (16) Å^3^
*Z* = 4
Mo *K*α radiation
μ = 1.82 mm^−1^
*T* = 200 K
0.58 × 0.16 × 0.07 mm

#### Data collection

Bruker APEXII CCD diffractometer
Absorption correction: multi-scan (*SADABS*; Bruker, 2008 ▶) *T*_min_ = 0.417, *T*_max_ = 0.889
22440 measured reflections
5775 independent reflections
3774 reflections with *I* > 2σ(*I*)
*R*_int_ = 0.030

#### Refinement

*R*[*F* ^2^ > 2σ(*F* ^2^)] = 0.051
*wR*(*F* ^2^) = 0.141
*S* = 1.02
5775 reflections
326 parameters
H-atom parameters constrained
Δρ_max_ = 0.61 e Å^−3^
Δρ_min_ = −0.93 e Å^−3^

### 

Data collection: *APEX2* (Bruker, 2010 ▶); cell refinement: *SAINT* (Bruker, 2010 ▶); data reduction: *SAINT*; program(s) used to solve structure: *SHELXS97* (Sheldrick, 2008 ▶); program(s) used to refine structure: *SHELXL97* (Sheldrick, 2008 ▶); molecular graphics: *ORTEP-3* (Farrugia, 2012 ▶) and *Mercury* (Macrae *et al.*, 2008 ▶); software used to prepare material for publication: *SHELXL97* and *PLATON* (Spek, 2009 ▶).

## Supplementary Material

Crystal structure: contains datablock(s) I, global. DOI: 10.1107/S1600536812046417/im2409sup1.cif

Supplementary material file. DOI: 10.1107/S1600536812046417/im2409Isup2.cdx

Structure factors: contains datablock(s) I. DOI: 10.1107/S1600536812046417/im2409Isup3.hkl

Supplementary material file. DOI: 10.1107/S1600536812046417/im2409Isup4.cml

Additional supplementary materials:  crystallographic information; 3D view; checkCIF report

## Figures and Tables

**Table 1:** Hydrogen-bond geometry (Å, °)

*D*—H⋯*A*	*D*—H	H⋯*A*	*D*⋯*A*	*D*—H⋯*A*
C2—H2*A*⋯N2^i^	0.99	2.62	3.339 (4)	129
C3—H3⋯N3^i^	0.95	2.65	3.288 (4)	125
C2—H2*B*⋯F2^ii^	0.99	2.45	3.308 (3)	144
C5—H5*A*⋯O3^iii^	0.99	2.37	3.258 (4)	149
C26—H26⋯O1^iv^	0.95	2.57	3.354 (5)	140

Symmetry codes: (i) 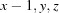; (ii) 
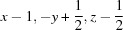
; (iii) 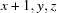; (iv) 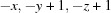.

**Fig. 1.**
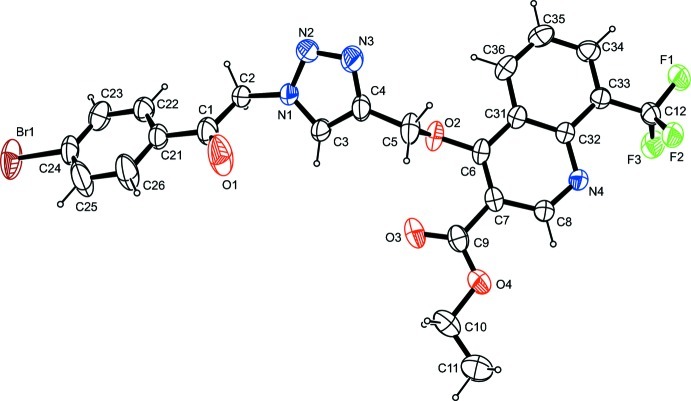
Molecular structure of the title compound, with anisotropic displacement ellipsoids drawn at the 50% probability level.

**Fig. 2.**
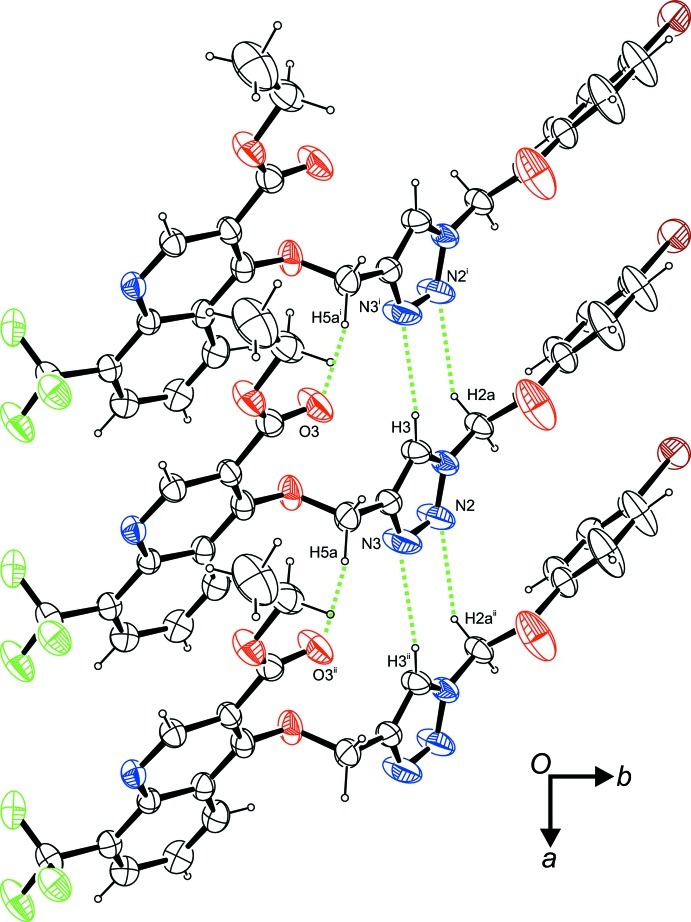
Intermolecular contacts, viewed along [0 0 - 1]. For reasons of clarity, only a selection of contacts is shown. Symmetry operators: ix - 1, y, z; iix + 1, y, z.

**Fig. 3.**
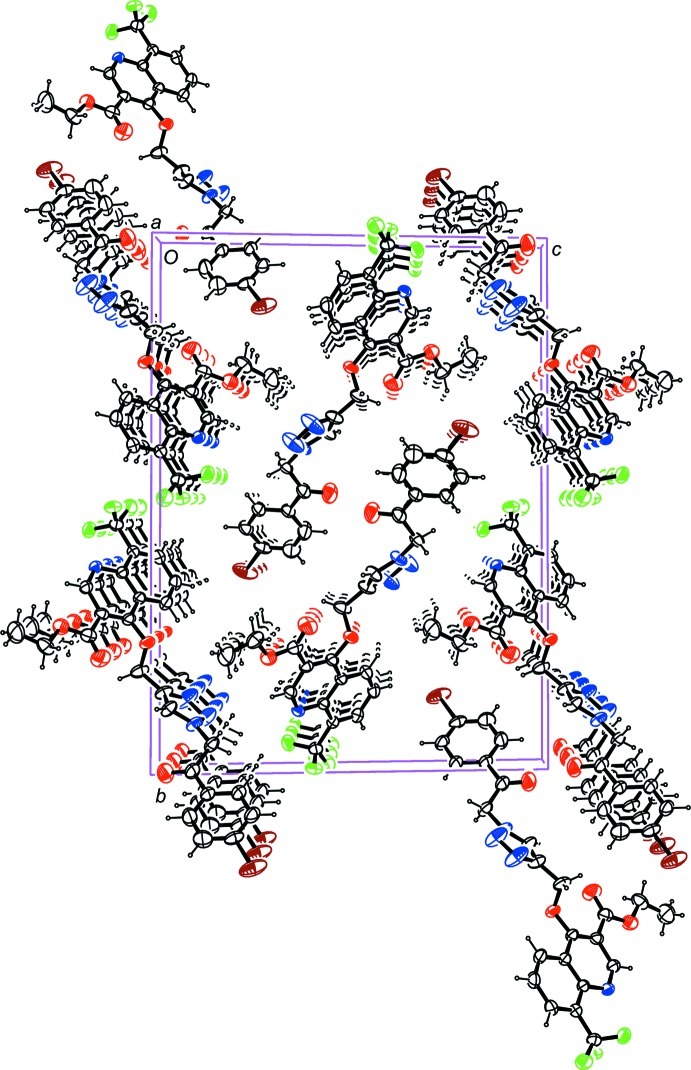
Molecular packing of the title compound, viewed along [-1 0 0] (anisotropic displacement ellipsoids drawn at the 50% probability level).

## supplementary crystallographic information

### Comment

Heterocyclic compounds have a wide range of applications in a vast variety of different fields such as pharmacy, agrochemistry and even in optoelectronics (Isloor *et al.*, 2009; Vijesh *et al.*, 2011; Ruanwasa *et al.*, 2010). Quinoline and its derivatives are well known nitrogen-containing heterocyclic compounds and play an important role in medicinal and pesticide chemistry by exhibiting a wide range of activities such as antibacterial, antifungal, antibiotic, anticancer, anticonvulsant, anti-tuberculosis and anti-inflammatory properties (Chen *et al.*, 2004; Kaur *et al.*, 2010; Bekhit *et al.* 2004). Keeping in mind the biological importance of quinoline-derived compounds, the title compound was synthesized to study its crystal structure.

The least-squares plane defined by the non-hydrogen atoms of the 1,2,3-triazole core encloses angles of 27.2 (2) ° and 48.4 (2) ° with the least-squares planes defined by the intracyclic atoms of the quinoline scaffold as well as the phenyl group, respectively. The latter two mentioned planes intersect at an angle of 41.5 (1) °. The quinoline scaffold is almost planar (r.m.s. of all fitted non-hydrogen atoms = 0.0176 Å) with C35 deviating most from this common plane by 0.028 (3) Å (Fig. 1).

In the crystal, intermolecular C–H···O, C–H···F and C–H···N contacts can be detected whose range falls by at least 0.1 Å below the sum of van-der-Waals radii of the atoms participating in them. The C–H···N contacts – whose angles fall markedly below a linear arrangement for the donor atom, hydrogen atom as well as the acceptor atom – likely are to be seen as a result of the more pronounced C–H···O contacts described below (*see below*). They are supported by one of the hydrogen atoms on one of the methylene groups directly bonded to the 1,2,3-triazole core as well as the latter one's intracyclic CH group and have the two two-coordinate nitrogen atoms as acceptors. These contacts form two homodromic chains connecting the molecules to chains along the crystallographic *a* axis. Along these chains, one set of C–H···O contacts between the second methylene group bonded to the 1,2,3-triazole core as well as the double bonded oxygen atom of the ester group can be found. The second set of C–H···O contacts is apparent between one of the hydrogen atoms on the bromophenyl group in *ortho* position to the keto group as donor and the keto group in a neighbouring molecule as acceptor. The intermolecular C–H···F contact is supported by the second hydrogen atom of the methylene group that is already part of the C–H···N contact system. In addition, an intramolecular C–H···F contact can be hold responsible for the small F–C–C–C_H_ dihedral angle that was measured at 0.6 (4) ° only. Metrical parameters as well as information about the symmetry of these contacts are summarized in Table 1. In total, these contacts connect the molecules to a three-dimensional network. According to a graph-set analysis (Etter *et al.*, 1990; Bernstein *et al.*, 1995), the descriptor for the C–H···N contacts is *C*^1^_1_(4)*C*^1^_1_(4) on the unary level while the C–H···O contacts require a *C*^1^_1_(7)*R*^2^_2_(10) descriptor on the same level. The descriptor for the C–H···F contacts is *S*(5)*C*^1^_1_(13). The shortest intercentroid distance between two aromatic systems was measured at 3.896 (2) Å and is found between the two different six-membered rings of the quinoline scaffold in neighbouring molecules.

The packing of the title compound in the crystal structure is shown in Figure 3.

### Experimental

To a stirred solution of 2-bromo-1-(4-bromophenyl)ethanone (0.50 g, 0.0017 mol), sodium azide (0.117 g, 0.0018 mol) in aqueous PEG 400 (5 ml, *v*:*v* = 1:1), ethyl 4-oxo-1-(prop-2-yn-1-yl)-8- (trifluoromethyl)-1,4-dihydroquinoline-3-carboxylate (0.58 g, 0.0018 mol), sodium ascorbate (0.356 g, 0.0018 mol) and 10 mol % of copper iodide were added. The heterogeneous mixture was stirred vigorously overnight. Completion of the reaction was monitored by TLC. The product was extracted in ethyl acetate and concentrated. The crude product was purified by column chromatography using petrol ether and ethyl acetate as the eluent, yield: 0.53 g (52.47%). Single crystals suitable for the X-ray diffraction study were obtained by slow evaporation of a solution of the compound in ethyl acetate at room temperature.

### Refinement

Carbon-bound H atoms were placed in calculated positions (C–H 0.95 Å for aromatic carbon atoms and C–H 0.99 Å for methylene groups) and were included in the refinement in the riding model approximation, with *U*_iso_(H) set to 1.2*U*_eq_(C). The H atoms of the methyl groups were allowed to rotate with a fixed angle around the C—C bond to best fit the experimental electron density (HFIX 137 in the *SHELX* program suite (Sheldrick, 2008)), with *U*_iso_(H) set to 1.5*U*_eq_(C).

### Crystal data

**Table:**

C_24_H_18_BrF_3_N_4_O_4_	*F*(000) = 1136
*M**_r_* = 563.33	*D*_x_ = 1.598 Mg m^−^^3^
Monoclinic, *P*2_1_/*c*	Melting point = 380–378 K
Hall symbol: -P 2ybc	Mo *K*α radiation, λ = 0.71073 Å
*a* = 5.2809 (2) Å	Cell parameters from 6880 reflections
*b* = 24.5131 (10) Å	θ = 2.4–27.9°
*c* = 18.3517 (7) Å	µ = 1.82 mm^−^^1^
β = 99.643 (1)°	*T* = 200 K
*V* = 2342.08 (16) Å^3^	Platelet, colourless
*Z* = 4	0.58 × 0.16 × 0.07 mm

### Data collection

**Table:**

Bruker APEXII CCD diffractometer	5775 independent reflections
Radiation source: fine-focus sealed tube	3774 reflections with *I* > 2σ(*I*)
Graphite monochromator	*R*_int_ = 0.030
φ and ω scans	θ_max_ = 28.3°, θ_min_ = 2.4°
Absorption correction: multi-scan (*SADABS*; Bruker, 2008)	*h* = −6→7
*T*_min_ = 0.417, *T*_max_ = 0.889	*k* = −32→32
22440 measured reflections	*l* = −24→24

### Refinement

**Table:**

Refinement on *F*^2^	Primary atom site location: structure-invariant direct methods
Least-squares matrix: full	Secondary atom site location: difference Fourier map
*R*[*F*^2^ > 2σ(*F*^2^)] = 0.051	Hydrogen site location: inferred from neighbouring sites
*wR*(*F*^2^) = 0.141	H-atom parameters constrained
*S* = 1.02	*w* = 1/[σ^2^(*F*_o_^2^) + (0.0546*P*)^2^ + 2.6613*P*] where *P* = (*F*_o_^2^ + 2*F*_c_^2^)/3
5775 reflections	(Δ/σ)_max_ = 0.001
326 parameters	Δρ_max_ = 0.61 e Å^−^^3^
0 restraints	Δρ_min_ = −0.93 e Å^−^^3^

### Fractional atomic coordinates and isotropic or equivalent isotropic displacement parameters (Å^2^)

**Table:**

	*x*	*y*	*z*	*U*_iso_*/*U*_eq_	
Br1	−0.68782 (8)	0.629887 (16)	0.22421 (3)	0.0859 (2)	
F1	1.3784 (4)	0.00897 (8)	0.56325 (11)	0.0659 (6)	
F2	1.3161 (4)	0.05001 (7)	0.66156 (9)	0.0494 (4)	
F3	1.0047 (4)	0.00785 (7)	0.59406 (11)	0.0550 (5)	
O1	0.2309 (7)	0.48213 (13)	0.44653 (15)	0.0921 (11)	
O2	0.6398 (4)	0.25396 (8)	0.50057 (11)	0.0477 (5)	
O3	0.3332 (5)	0.27788 (10)	0.60507 (14)	0.0658 (7)	
O4	0.3675 (5)	0.22131 (9)	0.70058 (13)	0.0549 (6)	
N1	0.4456 (5)	0.39894 (10)	0.38120 (14)	0.0440 (6)	
N2	0.6711 (6)	0.39092 (14)	0.3594 (2)	0.0709 (10)	
N3	0.8024 (6)	0.35804 (14)	0.4081 (2)	0.0763 (11)	
N4	0.8633 (5)	0.11738 (9)	0.63266 (12)	0.0349 (5)	
C1	0.1559 (7)	0.47765 (13)	0.38096 (18)	0.0505 (8)	
C2	0.2540 (6)	0.43291 (12)	0.33647 (16)	0.0433 (7)	
H2A	0.1080	0.4098	0.3139	0.052*	
H2B	0.3301	0.4495	0.2959	0.052*	
C3	0.4308 (6)	0.37070 (12)	0.44310 (17)	0.0442 (7)	
H3	0.2901	0.3692	0.4692	0.053*	
C4	0.6589 (6)	0.34487 (12)	0.46013 (18)	0.0450 (7)	
C5	0.7543 (6)	0.30655 (12)	0.52164 (19)	0.0500 (8)	
H5A	0.9440	0.3040	0.5290	0.060*	
H5B	0.7028	0.3193	0.5682	0.060*	
C6	0.7165 (6)	0.21159 (11)	0.54721 (15)	0.0370 (6)	
C7	0.6172 (5)	0.20139 (10)	0.61068 (15)	0.0353 (6)	
C8	0.7001 (6)	0.15307 (11)	0.65100 (15)	0.0366 (6)	
H8	0.6314	0.1462	0.6948	0.044*	
C9	0.4265 (6)	0.23796 (12)	0.63684 (17)	0.0417 (7)	
C10	0.1823 (7)	0.25434 (14)	0.73111 (19)	0.0539 (8)	
H10A	0.0194	0.2565	0.6956	0.065*	
H10B	0.2496	0.2918	0.7410	0.065*	
C11	0.1379 (11)	0.2287 (2)	0.7998 (2)	0.0903 (16)	
H11A	0.0886	0.1905	0.7903	0.135*	
H11B	−0.0002	0.2481	0.8186	0.135*	
H11C	0.2956	0.2305	0.8365	0.135*	
C12	1.2086 (6)	0.03989 (12)	0.59112 (16)	0.0423 (7)	
C21	−0.0461 (6)	0.51471 (12)	0.34071 (16)	0.0440 (7)	
C22	−0.1286 (6)	0.51169 (13)	0.26558 (16)	0.0451 (7)	
H22	−0.0565	0.4853	0.2372	0.054*	
C23	−0.3155 (7)	0.54683 (13)	0.23120 (18)	0.0513 (8)	
H23	−0.3684	0.5453	0.1791	0.062*	
C24	−0.4236 (6)	0.58361 (12)	0.2723 (2)	0.0508 (8)	
C25	−0.3501 (10)	0.58660 (17)	0.3464 (2)	0.0862 (15)	
H25	−0.4291	0.6121	0.3745	0.103*	
C26	−0.1584 (10)	0.55214 (17)	0.3812 (2)	0.0844 (15)	
H26	−0.1045	0.5544	0.4332	0.101*	
C31	0.8958 (5)	0.17471 (10)	0.52570 (14)	0.0347 (6)	
C32	0.9629 (5)	0.12778 (10)	0.56984 (14)	0.0311 (5)	
C33	1.1420 (5)	0.09074 (11)	0.54750 (14)	0.0341 (6)	
C34	1.2501 (6)	0.10091 (12)	0.48611 (15)	0.0410 (7)	
H34	1.3688	0.0756	0.4718	0.049*	
C35	1.1872 (7)	0.14842 (13)	0.44408 (16)	0.0470 (7)	
H35	1.2669	0.1554	0.4023	0.056*	
C36	1.0133 (6)	0.18436 (12)	0.46280 (16)	0.0446 (7)	
H36	0.9701	0.2161	0.4337	0.054*	

### Atomic displacement parameters (Å^2^)

**Table:**

	*U*^11^	*U*^22^	*U*^33^	*U*^12^	*U*^13^	*U*^23^
Br1	0.0515 (3)	0.0564 (2)	0.1386 (5)	0.01219 (17)	−0.0170 (2)	0.0347 (2)
F1	0.0879 (16)	0.0592 (12)	0.0565 (12)	0.0408 (11)	0.0290 (11)	0.0074 (9)
F2	0.0566 (12)	0.0539 (10)	0.0370 (9)	0.0161 (9)	0.0060 (8)	0.0067 (8)
F3	0.0720 (13)	0.0296 (8)	0.0640 (12)	−0.0009 (8)	0.0132 (10)	0.0037 (8)
O1	0.128 (3)	0.088 (2)	0.0487 (15)	0.061 (2)	−0.0188 (16)	−0.0027 (14)
O2	0.0599 (14)	0.0324 (10)	0.0452 (11)	0.0068 (9)	−0.0078 (10)	0.0091 (8)
O3	0.0578 (16)	0.0620 (15)	0.0772 (17)	0.0317 (13)	0.0103 (13)	0.0126 (13)
O4	0.0633 (16)	0.0503 (13)	0.0544 (14)	0.0210 (11)	0.0191 (11)	−0.0004 (10)
N1	0.0368 (14)	0.0421 (13)	0.0554 (15)	0.0115 (11)	0.0148 (12)	0.0196 (11)
N2	0.0441 (18)	0.080 (2)	0.097 (2)	0.0258 (15)	0.0346 (17)	0.0470 (19)
N3	0.0405 (18)	0.084 (2)	0.109 (3)	0.0245 (16)	0.0274 (18)	0.054 (2)
N4	0.0402 (14)	0.0305 (11)	0.0347 (12)	0.0042 (10)	0.0082 (10)	0.0039 (9)
C1	0.058 (2)	0.0491 (17)	0.0420 (17)	0.0170 (15)	0.0000 (15)	0.0087 (14)
C2	0.0420 (17)	0.0461 (16)	0.0434 (16)	0.0148 (13)	0.0117 (13)	0.0167 (13)
C3	0.0329 (16)	0.0486 (17)	0.0517 (18)	0.0087 (13)	0.0091 (13)	0.0211 (14)
C4	0.0335 (16)	0.0364 (14)	0.063 (2)	0.0054 (12)	0.0030 (14)	0.0159 (14)
C5	0.0422 (18)	0.0360 (15)	0.066 (2)	0.0021 (13)	−0.0089 (15)	0.0136 (14)
C6	0.0428 (16)	0.0296 (13)	0.0345 (14)	0.0034 (11)	−0.0057 (12)	0.0043 (10)
C7	0.0338 (15)	0.0299 (13)	0.0397 (15)	0.0049 (11)	−0.0016 (12)	−0.0018 (11)
C8	0.0402 (16)	0.0346 (13)	0.0354 (14)	0.0045 (12)	0.0079 (12)	0.0028 (11)
C9	0.0343 (16)	0.0373 (14)	0.0500 (18)	0.0035 (12)	−0.0032 (13)	−0.0038 (12)
C10	0.046 (2)	0.0561 (19)	0.059 (2)	0.0090 (15)	0.0088 (15)	−0.0173 (16)
C11	0.123 (4)	0.093 (3)	0.063 (3)	0.038 (3)	0.041 (3)	−0.002 (2)
C12	0.0488 (19)	0.0375 (15)	0.0416 (16)	0.0141 (13)	0.0103 (13)	0.0011 (12)
C21	0.0514 (19)	0.0389 (15)	0.0404 (16)	0.0131 (13)	0.0038 (13)	0.0070 (12)
C22	0.0502 (19)	0.0439 (16)	0.0394 (16)	0.0094 (14)	0.0028 (13)	0.0052 (12)
C23	0.056 (2)	0.0464 (17)	0.0446 (17)	−0.0030 (15)	−0.0119 (14)	0.0093 (14)
C24	0.0431 (18)	0.0360 (15)	0.070 (2)	0.0113 (13)	−0.0011 (15)	0.0160 (15)
C25	0.116 (4)	0.070 (3)	0.070 (3)	0.061 (3)	0.009 (3)	0.001 (2)
C26	0.132 (4)	0.076 (3)	0.0401 (19)	0.063 (3)	−0.001 (2)	−0.0019 (17)
C31	0.0400 (15)	0.0307 (12)	0.0314 (13)	−0.0003 (11)	0.0002 (11)	0.0019 (10)
C32	0.0324 (14)	0.0285 (12)	0.0311 (13)	−0.0007 (10)	0.0019 (11)	0.0013 (10)
C33	0.0384 (15)	0.0325 (13)	0.0311 (13)	0.0013 (11)	0.0047 (11)	−0.0015 (10)
C34	0.0465 (18)	0.0437 (15)	0.0341 (14)	0.0009 (13)	0.0102 (13)	−0.0050 (12)
C35	0.059 (2)	0.0517 (17)	0.0318 (15)	−0.0047 (15)	0.0123 (14)	0.0011 (13)
C36	0.056 (2)	0.0419 (15)	0.0340 (15)	−0.0027 (14)	0.0031 (13)	0.0090 (12)

### Geometric parameters (Å, º)

**Table:**

Br1—C24	1.897 (3)	C7—C9	1.487 (4)
F1—C12	1.339 (3)	C8—H8	0.9500
F2—C12	1.345 (3)	C10—C11	1.461 (6)
F3—C12	1.341 (4)	C10—H10A	0.9900
O1—C1	1.207 (4)	C10—H10B	0.9900
O2—C6	1.364 (3)	C11—H11A	0.9800
O2—C5	1.448 (4)	C11—H11B	0.9800
O3—C9	1.202 (4)	C11—H11C	0.9800
O4—C9	1.324 (4)	C12—C33	1.491 (4)
O4—C10	1.452 (4)	C21—C26	1.376 (5)
N1—N2	1.333 (4)	C21—C22	1.377 (4)
N1—C3	1.344 (4)	C22—C23	1.380 (4)
N1—C2	1.453 (3)	C22—H22	0.9500
N2—N3	1.311 (4)	C23—C24	1.361 (5)
N3—C4	1.354 (4)	C23—H23	0.9500
N4—C8	1.311 (4)	C24—C25	1.353 (5)
N4—C32	1.369 (4)	C25—C26	1.389 (5)
C1—C21	1.498 (4)	C25—H25	0.9500
C1—C2	1.510 (4)	C26—H26	0.9500
C2—H2A	0.9900	C31—C32	1.417 (3)
C2—H2B	0.9900	C31—C36	1.419 (4)
C3—C4	1.351 (4)	C32—C33	1.420 (4)
C3—H3	0.9500	C33—C34	1.368 (4)
C4—C5	1.490 (4)	C34—C35	1.406 (4)
C5—H5A	0.9900	C34—H34	0.9500
C5—H5B	0.9900	C35—C36	1.358 (5)
C6—C7	1.378 (4)	C35—H35	0.9500
C6—C31	1.413 (4)	C36—H36	0.9500
C7—C8	1.426 (4)		
			
C6—O2—C5	116.3 (2)	C10—C11—H11B	109.5
C9—O4—C10	116.2 (2)	H11A—C11—H11B	109.5
N2—N1—C3	111.0 (2)	C10—C11—H11C	109.5
N2—N1—C2	119.2 (2)	H11A—C11—H11C	109.5
C3—N1—C2	129.7 (3)	H11B—C11—H11C	109.5
N3—N2—N1	106.5 (3)	F1—C12—F3	106.1 (2)
N2—N3—C4	109.4 (3)	F1—C12—F2	105.6 (2)
C8—N4—C32	117.0 (2)	F3—C12—F2	106.4 (2)
O1—C1—C21	121.6 (3)	F1—C12—C33	112.4 (2)
O1—C1—C2	121.3 (3)	F3—C12—C33	113.1 (2)
C21—C1—C2	117.0 (3)	F2—C12—C33	112.7 (2)
N1—C2—C1	112.4 (3)	C26—C21—C22	118.9 (3)
N1—C2—H2A	109.1	C26—C21—C1	118.3 (3)
C1—C2—H2A	109.1	C22—C21—C1	122.8 (3)
N1—C2—H2B	109.1	C21—C22—C23	120.4 (3)
C1—C2—H2B	109.1	C21—C22—H22	119.8
H2A—C2—H2B	107.9	C23—C22—H22	119.8
N1—C3—C4	105.1 (3)	C24—C23—C22	119.6 (3)
N1—C3—H3	127.4	C24—C23—H23	120.2
C4—C3—H3	127.4	C22—C23—H23	120.2
C3—C4—N3	107.9 (3)	C25—C24—C23	121.1 (3)
C3—C4—C5	130.3 (3)	C25—C24—Br1	120.0 (3)
N3—C4—C5	121.8 (3)	C23—C24—Br1	118.8 (3)
O2—C5—C4	106.6 (2)	C24—C25—C26	119.6 (3)
O2—C5—H5A	110.4	C24—C25—H25	120.2
C4—C5—H5A	110.4	C26—C25—H25	120.2
O2—C5—H5B	110.4	C21—C26—C25	120.3 (3)
C4—C5—H5B	110.4	C21—C26—H26	119.9
H5A—C5—H5B	108.6	C25—C26—H26	119.9
O2—C6—C7	123.6 (3)	C6—C31—C32	118.3 (2)
O2—C6—C31	117.0 (3)	C6—C31—C36	121.7 (2)
C7—C6—C31	119.2 (2)	C32—C31—C36	119.9 (3)
C6—C7—C8	117.5 (2)	N4—C32—C31	122.6 (2)
C6—C7—C9	122.5 (2)	N4—C32—C33	119.5 (2)
C8—C7—C9	119.9 (3)	C31—C32—C33	117.9 (2)
N4—C8—C7	125.4 (3)	C34—C33—C32	120.7 (2)
N4—C8—H8	117.3	C34—C33—C12	120.1 (3)
C7—C8—H8	117.3	C32—C33—C12	119.2 (2)
O3—C9—O4	122.8 (3)	C33—C34—C35	120.7 (3)
O3—C9—C7	125.4 (3)	C33—C34—H34	119.7
O4—C9—C7	111.8 (2)	C35—C34—H34	119.7
O4—C10—C11	108.0 (3)	C36—C35—C34	120.4 (3)
O4—C10—H10A	110.1	C36—C35—H35	119.8
C11—C10—H10A	110.1	C34—C35—H35	119.8
O4—C10—H10B	110.1	C35—C36—C31	120.3 (3)
C11—C10—H10B	110.1	C35—C36—H36	119.9
H10A—C10—H10B	108.4	C31—C36—H36	119.9
C10—C11—H11A	109.5		
			
C3—N1—N2—N3	−0.8 (5)	C26—C21—C22—C23	2.0 (6)
C2—N1—N2—N3	−178.3 (3)	C1—C21—C22—C23	180.0 (3)
N1—N2—N3—C4	0.6 (5)	C21—C22—C23—C24	−1.9 (5)
N2—N1—C2—C1	−127.2 (3)	C22—C23—C24—C25	0.4 (6)
C3—N1—C2—C1	55.8 (5)	C22—C23—C24—Br1	−178.0 (3)
O1—C1—C2—N1	−1.4 (5)	C23—C24—C25—C26	1.0 (7)
C21—C1—C2—N1	−179.0 (3)	Br1—C24—C25—C26	179.3 (4)
N2—N1—C3—C4	0.7 (4)	C22—C21—C26—C25	−0.6 (7)
C2—N1—C3—C4	177.9 (3)	C1—C21—C26—C25	−178.7 (4)
N1—C3—C4—N3	−0.3 (4)	C24—C25—C26—C21	−0.8 (8)
N1—C3—C4—C5	−178.3 (3)	O2—C6—C31—C32	−174.8 (2)
N2—N3—C4—C3	−0.2 (5)	C7—C6—C31—C32	1.3 (4)
N2—N3—C4—C5	178.1 (3)	O2—C6—C31—C36	6.8 (4)
C6—O2—C5—C4	175.5 (3)	C7—C6—C31—C36	−177.1 (3)
C3—C4—C5—O2	78.5 (5)	C8—N4—C32—C31	0.0 (4)
N3—C4—C5—O2	−99.2 (4)	C8—N4—C32—C33	179.6 (3)
C5—O2—C6—C7	81.6 (4)	C6—C31—C32—N4	−0.9 (4)
C5—O2—C6—C31	−102.5 (3)	C36—C31—C32—N4	177.5 (3)
O2—C6—C7—C8	175.0 (3)	C6—C31—C32—C33	179.6 (2)
C31—C6—C7—C8	−0.9 (4)	C36—C31—C32—C33	−2.1 (4)
O2—C6—C7—C9	−3.8 (4)	N4—C32—C33—C34	−178.1 (3)
C31—C6—C7—C9	−179.6 (3)	C31—C32—C33—C34	1.4 (4)
C32—N4—C8—C7	0.5 (4)	N4—C32—C33—C12	2.8 (4)
C6—C7—C8—N4	0.0 (4)	C31—C32—C33—C12	−177.6 (3)
C9—C7—C8—N4	178.7 (3)	F1—C12—C33—C34	0.6 (4)
C10—O4—C9—O3	−0.3 (5)	F3—C12—C33—C34	−119.5 (3)
C10—O4—C9—C7	179.9 (3)	F2—C12—C33—C34	119.8 (3)
C6—C7—C9—O3	3.1 (5)	F1—C12—C33—C32	179.7 (3)
C8—C7—C9—O3	−175.6 (3)	F3—C12—C33—C32	59.6 (3)
C6—C7—C9—O4	−177.2 (3)	F2—C12—C33—C32	−61.1 (4)
C8—C7—C9—O4	4.1 (4)	C32—C33—C34—C35	0.4 (4)
C9—O4—C10—C11	178.5 (3)	C12—C33—C34—C35	179.4 (3)
O1—C1—C21—C26	−4.3 (6)	C33—C34—C35—C36	−1.6 (5)
C2—C1—C21—C26	173.3 (4)	C34—C35—C36—C31	0.9 (5)
O1—C1—C21—C22	177.7 (4)	C6—C31—C36—C35	179.3 (3)
C2—C1—C21—C22	−4.7 (5)	C32—C31—C36—C35	0.9 (4)

### Hydrogen-bond geometry (Å, º)

**Table:**

*D*—H···*A*	*D*—H	H···*A*	*D*···*A*	*D*—H···*A*
C2—H2*A*···N2^i^	0.99	2.62	3.339 (4)	129
C3—H3···N3^i^	0.95	2.65	3.288 (4)	125
C2—H2*B*···F2^ii^	0.99	2.45	3.308 (3)	144
C5—H5*A*···O3^iii^	0.99	2.37	3.258 (4)	149
C26—H26···O1^iv^	0.95	2.57	3.354 (5)	140
C34—H34···F1	0.95	2.34	2.687 (4)	101

Symmetry codes: (i) *x*−1, *y*, *z*; (ii) *x*−1, −*y*+1/2, *z*−1/2; (iii) *x*+1, *y*, *z*; (iv) −*x*, −*y*+1, −*z*+1.
